# Steppogenin Isolated from *Cudrania tricuspidata* Shows Antineuroinflammatory Effects via NF-κB and MAPK Pathways in LPS-Stimulated BV2 and Primary Rat Microglial Cells

**DOI:** 10.3390/molecules22122130

**Published:** 2017-12-02

**Authors:** Dong-Cheol Kim, Tran Hong Quang, Hyuncheol Oh, Youn-Chul Kim

**Affiliations:** 1 Institute of Pharmaceutical Research and Development, College of Pharmacy, Wonkwang University, Iksan 54538, Korea; kimman07@hanmail.net (D.-C.K.); hoh@wku.ac.kr (H.O.); 2 Institute of Marine Biochemistry, Vietnam Academy of Science and Technology (VAST), 18 Hoang Quoc Viet, Caugiay, Hanoi 122102, Vietnam; quangth2004@yahoo.com; 3 Hanbang Cardio-Renal Syndrome Research Center, Wonkwang University, Iksan 54538, Korea

**Keywords:** steppogenin, *Cudrania tricuspidata*, neuroinflammation, nuclear factor-kappa B (NF-κB), mitogen-activated protein kinase (MAPK)

## Abstract

Excessive microglial stimulation has been recognized in several neurodegenerative diseases, including Parkinson’s disease (PD), Alzheimer’s disease (AD), amyotropic lateral sclerosis (ALS), HIV-associated dementia (HAD), multiple sclerosis (MS), and stroke. When microglia are stimulated, they produce proinflammatory mediators and cytokines, including nitric oxide (NO) derived from inducible NO synthase (iNOS), prostaglandin E2 (PGE_2_) derived from cyclooxygenase-2 (COX-2), tumor necrosis factor-α (TNF-α), interleukin-1β (IL-1β), interleukin-12 (IL-12), and interleukin-6 (IL-6). These inflammatory reactions are related to the nuclear factor-kappa B (NF-κB) and mitogen-activated protein kinase (MAPK) signaling pathways. Therefore, the modulation of NF-κB and MAPK is vital to prevent microglial activation and confer resistance against neuronal injury. In this study, steppogenin (**1**) isolated from *Cudrania tricuspidata* suppressed the neuroinflammatory responses to lipopolysaccharide (LPS). Steppogenin (**1**) inhibited the production of proinflammatory mediators and cytokines in LPS-challenged BV2 and rat primary microglial cells. Moreover, western blot analysis and immunofluorescence revealed that the nuclear translocation of NF-κB was inhibited in LPS-induced BV2 and rat primary microglial cells. The LPS-stimulated activation of BV2 and rat primary microglial cells was inhibited by steppogenin (**1**) through the suppression of c-Jun NH2-terminal kinase (JNK) and p38 MAPK signaling. These results suggested that steppogenin (**1**) exerted antineuroinflammatory effects against acute neuroinflammation in BV2 and rat primary microglial cells by suppressing the activation of NF-κB and MAPK signaling and the production of proinflammatory mediators and cytokines.

## 1. Introduction

*Cudrania tricuspidata*, a member of the *Moraceae* family, is a deciduous broad-leaved thorny tree grown throughout East Asia in Korea, China, and Japan. In Korean traditional medicine, the *C. tricuspidata* has been used to treat impotency, insomnia, and poor health [1]. Moreover, *C. tricuspidata* root bark and bark have been used in oriental medicine to treat neuritis and inflammation [2]. *C. tricuspidata* contains a variety of components, including flavonoids [3], glycoproteins [4], and xanthones [5]. In recent studies of the pharmacological effects of *C. tricuspidata*, the extracts have been shown to possess a variety of biological effects, including antioxidant [6], hepatoprotective [7], neuroprotective [8], monoamine oxidase-A inhibitory [9], antiatherosclerotic, and anti-inflammatory activities [10].

Microglial cells, considered to be the macrophages of the central nervous system (CNS), play a vital role in neuronal recovery and normal brain development. The overactivation of microglia cells leads to neuronal injury and the release of neurotoxic factors, including prostaglandin E2 (PGE_2_) and nitric oxide (NO), as well as inflammatory mediators and cytokines, such as tumor necrosis factor-α (TNF-α), interleukin-1β (IL-1β), interleukin-12 (IL-12), and interleukin-6 (IL-6), which are involved in neurodegenerative diseases [11,12,13]. BV2 cells, immortalized microglial cells, are commonly used instead of primary microglial cells in in vitro inflammation models [14]. The nuclear factor-kappa B (NF-κB) signaling pathway is activated in these cells when exposed to lipopolysaccharide (LPS). LPS is a prominent cell wall component of Gram-negative bacteria, which is a strong activator of microglial cells [15]. In microglia activated by LPS, NF-κB is unbound from the inhibitor κB (IκB)-α. The free NF-κB heterodimer components, p50 and p65, translocate into the nucleus and bind to the kappaB (κB) site. Moreover, NF-κB transcripts regulate inflammatory mediators, cytokines, and proteins, such as inducible nitric oxide synthase (iNOS), cyclooxygenase-2 (COX-2) [16,17]. NF-κB and mitogen-activated protein kinases (MAPKs) play important roles in inflammatory response. MAPKs are intracellular serine/threonine protein kinases that consist of extracellular signal-regulated kinase 1/2 (ERK1/2), p38, and c-Jun NH2-terminal kinase (JNK) [18]. The involvement of MAPKs in various cellular procedures, such as proliferation, cell growth, cell death, differentiation, and immune responses, has been demonstrated. JNK and p38 are vital components of the MAPK, which are related to inflammatory reactions [19].

In the present study, we investigated an antineuroinflammatory reaction in the NF-κB and MAPK signaling pathway after treatment with steppogenin (**1**) in LPS-stimulated immortalized BV2 and rat primary microglial cells. 

## 2. Results

### 2.1. Chemical Structure of Steppogenin *(**1**)* and Its Effects on the Viability of BV2 Microglial Cells

**Figure 1:**
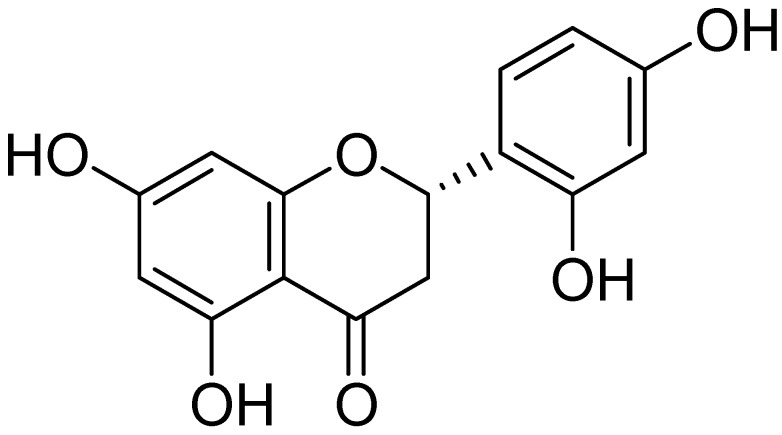
Chemical structure of steppogenin (**1**).

**Figure 2:**
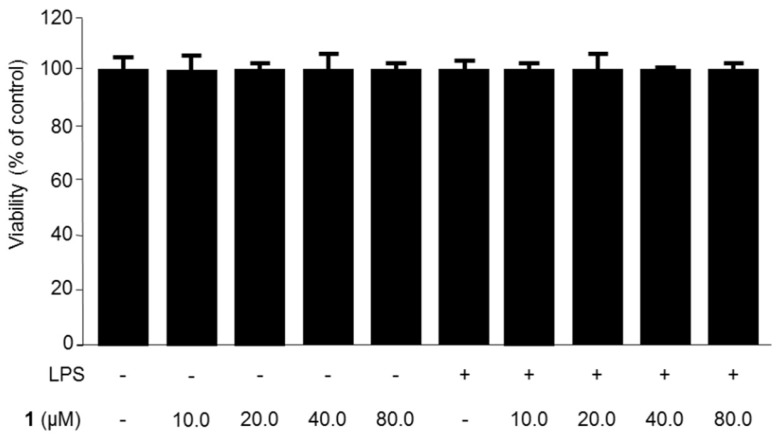
The effects of steppogenin (**1**) on the cell viability of BV2 microglial cells. BV2 microglial cells were incubated for 24 h with steppogenin in the range from 10.0 to 80.0 μM. The data are presented as the mean ± SD of three experiments.

The isolation of steppogenin (**1**) from *Cudrania tricuspidata* (Figure 1) has been described in our previous study [7]. To determine the cytotoxic effects of steppogenin (**1**), we performed 3-(4,5-dimethylthiazol-2-yl)-2,5-diphenyltetrazolium bromide (MTT) assay, but observed no cytotoxicity when the cells were treated with between 10.0 and 80.0 μM of **1** (Figure 2).

### 2.2. Effects of Steppogenin *(**1**)* on the mRNA Expression of the Proinflammatory Cytokines TNF-α, IL-1β, IL-12, and IL-6 in LPS-Stimulated BV2 Microglial Cells

**Figure 3:**
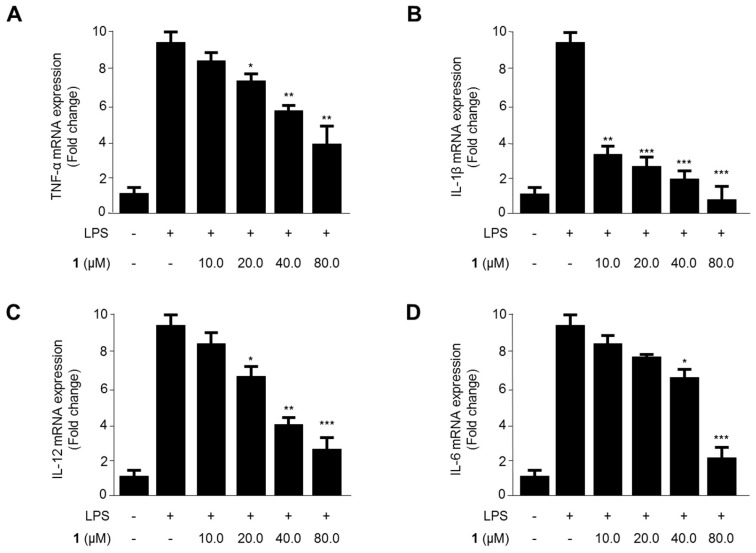
The effects of steppogenin (**1**) on the mRNA expression of tumor necrosis factor (TNF)-α (**A**), interleukin (IL)-1β (**B**), IL-12 (**C**), and IL-6 (**D**) in lipopolysaccharide (LPS)-stimulated BV2 microglial cells. (**A**–**D**) The cells were pretreated for 3 h with the indicated concentrations of **1** and then stimulated for 12 h with LPS (1 μg/mL). The data are presented as the mean ± SD of three experiments. * *p* < 0.05; ** *p* < 0.01; *** *p* < 0.001 compared with the LPS-treated group.

We evaluated the effects of steppogenin (**1**) on the mRNA expression of TNF-α, IL-1β, IL-12, and IL-6 in LPS-treated BV2 microglial cells (Figure 3). The levels of proinflammatory cytokines reduced after exposure to 10.0–80.0 μM steppogenin (**1**) for 12 h in LPS-treated BV2 microglial cells. As shown in Figure 3A–D, steppogenin (**1**) reduced the expression of TNF-α, IL-1β, IL-12, and IL-6 in a dose-dependent manner, as measured by quantitative real-time reverse transcriptase polymerase chain reaction (PCR).

### 2.3. Effects of Steppogenin *(**1**)* on Nitrite and PGE_2_ Production and iNOS and COX-2 Protein expression in LPS-Stimulated BV2 Microglial Cells

**Figure 4:**
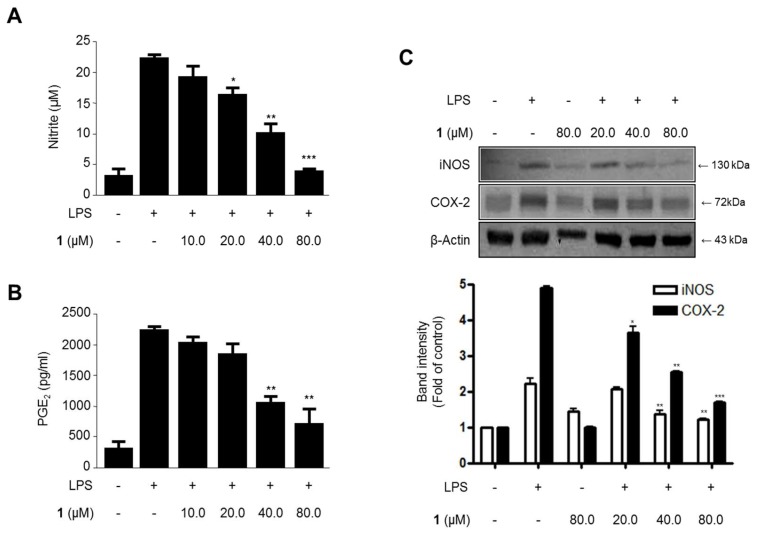
The effects of steppogenin (**1**) on nitrite (**A**) and prostaglandin E2 (PGE_2_) (**B**) production and iNOS and COX-2 expression (**C**) in lipopolysaccharide (LPS)-stimulated BV2 microglial cells. (**A**–**C**) The cells were pretreated for 3 h with the indicated concentrations of **1** and then stimulated for 24 h with LPS (1 μg/mL). The data are presented as the mean ± SD of three experiments. The band intensity was quantified by densitometry and normalized to the intensity of the β-actin band; the normalized values are presented below each band. * *p* < 0.05; ** *p* < 0.01; *** *p* < 0.001 compared with the LPS-treated group.

To investigate the effects of steppogenin (**1**) on LPS-induced nitrite and PGE_2_ production and iNOS and COX-2 protein expression (Figure 4), the cells were treated with or without LPS (1 μg/mL) in the presence or absence of steppogenin (**1**) for 24 h. The upregulation of nitrite (Figure 4A) and PGE_2_ (Figure 4B) production and iNOS and COX-2 protein expression (Figure 4C) were significantly inhibited by steppogenin (**1**) in dose-dependent manner. 

### 2.4. Effects of Steppogenin *(**1**)* on IκB-α Levels, NF-κB Nuclear Translocation, and NF-κB DNA Binding Activity in LPS-Stimulated BV2 Microglial Cells

**Figure 5:**
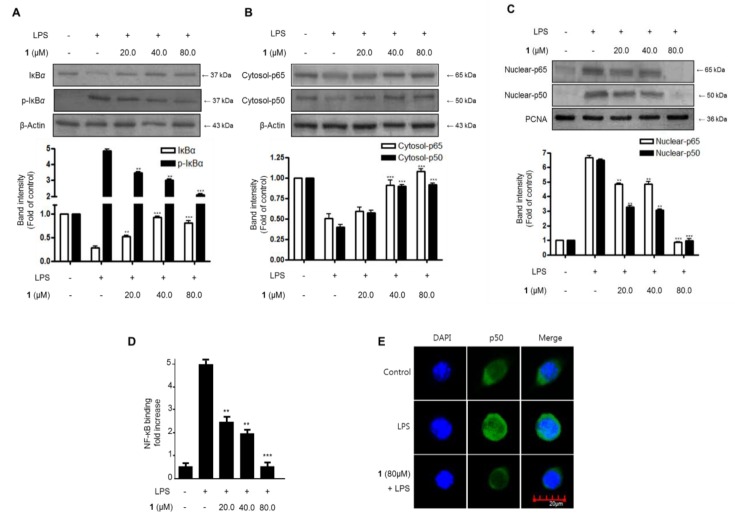
The effects of steppogenin (**1**) on IκB-α phosphorylation and degradation (**A**) NF-κB activation (**B**,**C**), NF-κB DNA binding activity (**D**), and NF-κB localization (**E**) in LPS-stimulated BV2 microglial cells. (**A**–**E**) The cells were pretreated for 3 h with the indicated concentrations of **1** and then stimulated for 1 h with LPS (1 μg/mL). The data are presented as the mean ± SD of three experiments. The band intensity was quantified by densitometry and normalized to the intensity of the β-actin or proliferating cell nuclear antigen (PCNA) band; the normalized values are presented below each band. ** *p* < 0.01; *** *p* < 0.001 compared with the LPS-treated group.

The effects of steppogenin (**1**) on the NF-κB (p50 and p65) pathway in LPS-challenged BV2 microglial cells were evaluated to investigate whether it regulated the transcription of inflammatory genes. First, we investigated the inhibitory effects on IκB-α degradation and phosphorylation. Next, we evaluated the inhibition of NF-κB (p50 and p65) nuclear translocation. As shown in Figure 5A, IκB-α was degraded after the exposure of BV2 microglia cells to LPS for 1 h. However, steppogenin (**1**) pretreatment (20.0–80.0 μM) significantly inhibited the phosphorylation of IκB-α (Figure 5A) in LPS-stimulated BV2 microglial cells. We then determined the effect of steppogenin (**1**) on the translocation of NF-κB in LPS-induced BV2 microglial cells. NF-κB translocation was blocked in steppogenin (**1**)-treated BV2 microglial cells (Figure 5B,C). We also evaluated the NF-κB DNA binding activity in the nuclear extracts of LPS-treated BV2 microglial cells. The DNA binding activity of NF-κB was increased by approximately 10-fold after LPS treatment, but this was suppressed by steppogenin (**1**) in a dose-dependent manner (Figure 5D). The confocal microscopic analysis indicated that the NF-κB/p50 protein was mostly present in the cytoplasm of unstimulated BV2 microglial cells. After treatment with LPS, NF-κB/p50 was observed to be translocated into the nucleus (Figure 5E).

### 2.5. Effects of Steppogenin *(**1**)* on the Phosphorylation of MAPKs in BV2 Microglial Cells Stimulated with LPS

**Figure 6:**
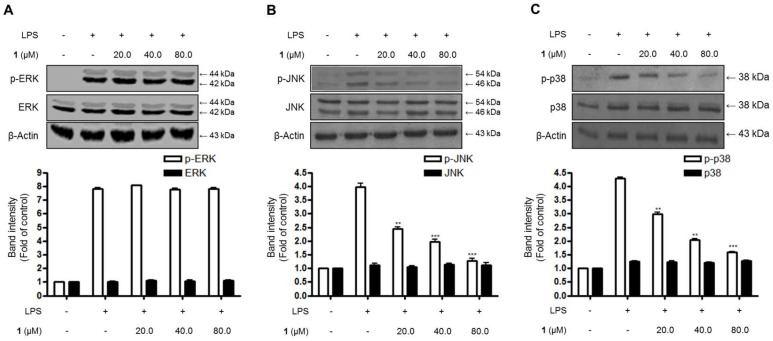
The effects of steppogenin (**1**) on extracellular signal–regulated kinase (ERK) (**A**), c-Jun N-terminal kinase (JNK) (**B**), and p38 (**C**) MAPK phosphorylation and protein expression. (**A**–**C**) The cells were pretreated for 3 h with the indicated concentrations of **1** and stimulated for 1 h with LPS (1 μg/mL). The levels of phosphorylated ERK (p-ERK), phosphorylated JNK (p-JNK), and phosphorylated-p38 MAPK (p-p38 MAPK) were determined by western blot analysis. Representative blots from three independent experiments with similar results and densitometric evaluations are shown. The band intensities were quantified by densitometry and normalized to the density of the β-actin band; the normalized values are presented below each band.

Additionally, the effects of steppogenin (**1**) on the stimulation of intracellular kinases, such as MAPKs, were examined in BV2 microglial cells. As shown in Figure 6, the phosphorylation of extracellular signal–regulated kinase (ERK), c-Jun N-terminal kinase (JNK), and p38 increased after treatment with LPS for 1 h in BV2 microglial cells. Moreover, 20.0–80.0 μM steppogenin (**1**) appeared to inhibit JNK and p38 MAPK phosphorylation in a dose-dependent manner (Figure 6B,C). Consequently, JNK and p38 MAPK phosphorylation were associated with the suppression of steppogenin (**1**) on LPS-stimulated NF-κB activation in LPS-challenged BV2 microglial cells.

### 2.6. Effects of Steppogenin *(**1**)* on Nitrite Production and iNOS and COX-2 Protein Expression in LPS-Stimulated Rat Primary Microglial Cells

**Figure 7:**
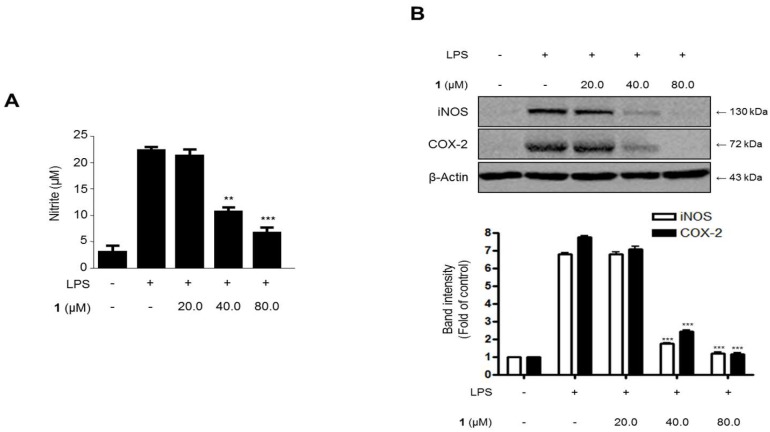
The effects of steppogenin (**1**) on nitrite (**A**) production and iNOS and COX-2 expression (**B**) in lipopolysaccharide (LPS)-stimulated primary rat microglial cells. (**A**,**B**) The cells were pretreated for 3 h with the indicated concentrations of **1** and then stimulated for 24 h with LPS (1 μg/mL). The data are presented as the mean ± SD of three experiments. The band intensities were quantified by densitometry and normalized to the intensities of the β-actin band; the normalized values are presented below each band. ** *p* < 0.01; *** *p* < 0.001 compared with the LPS-treated group.

To investigate the effects of steppogenin (**1**) on the LPS-challenged nitrite production and iNOS and COX-2 protein expression in rat primary microglial cells (Figure 7), the cells were treated with or without LPS (1 μg/mL) in the presence or absence of steppogenin (**1**) for 24 h. The upregulation in nitrite production (Figure 7A) and iNOS and COX-2 protein expression (Figure 7B) were significantly inhibited by steppogenin (**1**) in a dose-dependent manner. 

### 2.7. Effects of Steppogenin *(**1**)* on mRNA Expression of the Proinflammatory Cytokines TNF-α, IL-1β, IL-6, and IL-12 in LPS-Stimulated Rat Primary Microglial Cells

**Figure 8:**
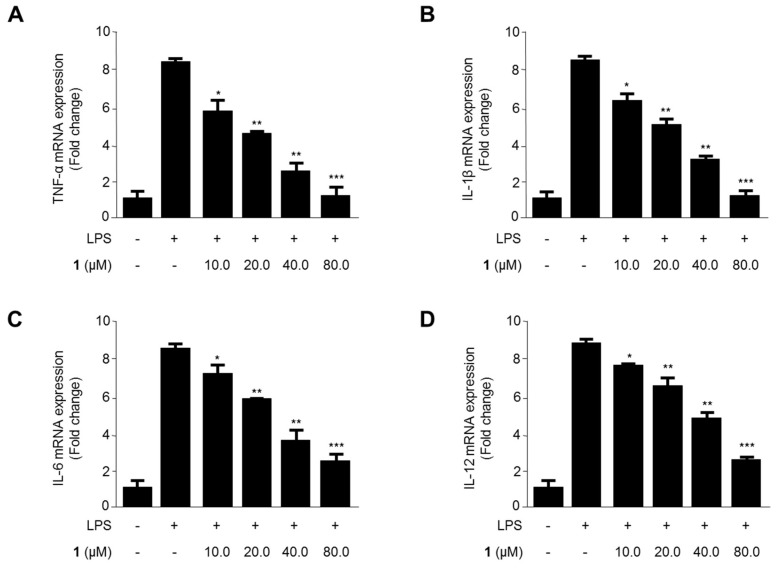
The effects of steppogenin (**1**) on the mRNA expression of TNF-α (**A**), IL-1β (**B**), IL-6 (**C**), and IL-12 (**D**) in LPS-stimulated primary rat microglial cells. (**A**–**D**) The cells were pretreated for 3 h with the indicated concentrations of **1** and then stimulated for 12 h with LPS (1 μg/mL). The data are presented as the mean ± SD of three experiments. * *p* < 0.05; ** *p* < 0.01; *** *p* < 0.001 compared with the LPS-treated group.

The effects of steppogenin (**1**) on the mRNA expression of TNF-α, IL-1β, IL-6, and IL-12 in LPS-treated rat primary microglial cells were evaluated (Figure 8). The mRNA expression of proinflammatory cytokines reduced when the LPS-treated cells were exposed to 10.0–80.0 μM steppogenin (**1**) for 12 h. As shown in Figure 8A–D, steppogenin (**1**) reduced the mRNA expression of TNF-α, IL-1β, IL-6, and IL-12 in a dose-dependent manner, as measured by quantitative real-time reverse transcriptase PCR.

### 2.8. Effects of Steppogenin *(**1**)* on IκB-α Levels, NF-κB Nuclear Translocation, and NF-κB DNA Binding Activity in LPS-Stimulated Rat Primary Microglial Cells

**Figure 9:**
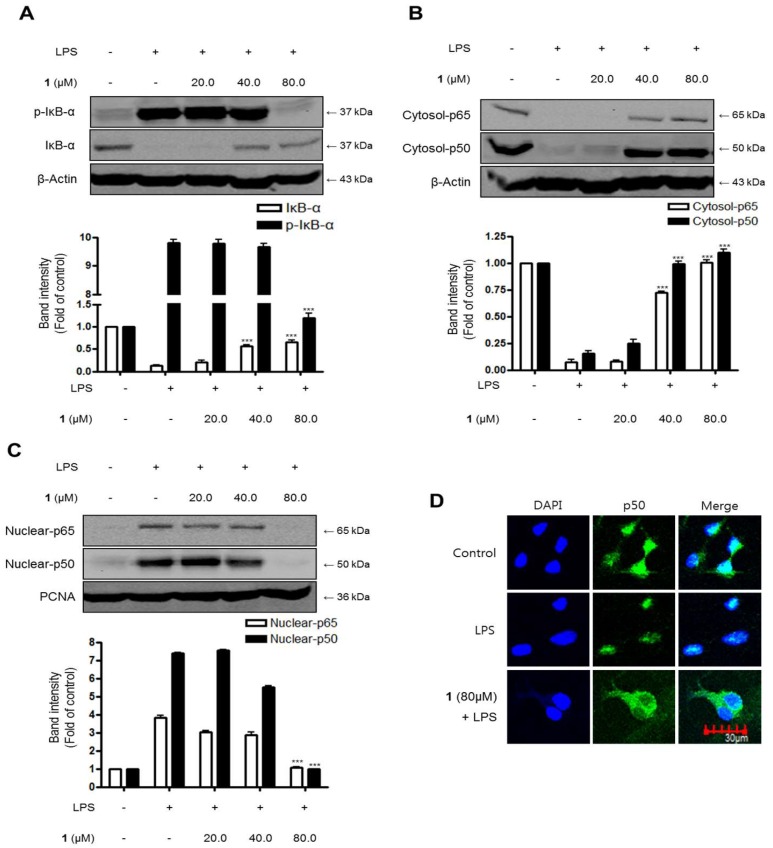
The effects of steppogenin (**1**) on IκB-α phosphorylation and degradation (**A**), NF-κB activation (**B**,**C**), and NF-κB localization (**D**) in LPS-stimulated primary rat microglial cells. (**A**–**D**) The cells were pretreated for 3 h with the indicated concentrations of **1** and then stimulated for 1 h with LPS (1 μg/mL). Total proteins were prepared and the western blot analysis was performed using specific IκB-α, p-IκB-α p65, and p50 antibodies. A commercially available NF-κB ELISA (Active Motif) was used to test the nuclear extracts and determine the degree of NF-κB binding. The data are presented as the mean ± SD of three experiments. The band intensities were quantified by densitometry and normalized to the intensity of β-actin or PCNA; the normalized values are presented below each band.

We investigated the inhibitory effects of steppogenin (**1**) on IκB-α degradation and phosphorylation and NF-κB (p50 and p65) nuclear translocationin LPS-stimulated rat primary microglial cells. As shown in Figure 9A, IκB-α was degraded after the exposure of BV2 microglia cells to LPS for 1 h. However, 20.0–80.0 μM steppogenin (**1**) pretreatment significantly repressed at 80 μM the phosphorylation of IκB-α (Figure 9A) in LPS-stimulated rat primary microglial cells. NF-κB translocation was blocked when rat primary microglial cells (Figure 9B,C) were treated with steppogenin (**1**). Confocal microscopic analysis indicated that the NF-κB/p50 protein was mostly present in the cytoplasm of unstimulated rat primary microglial cells. After treatment with LPS, translocated NF-κB/p50 was observed in the nucleus. However, steppogenin (**1**) inhibited the translocation of NF-κB/p50 protein like control levels (Figure 9D).

## 3. Discussion

An increasing body of demonstration appears that microglial cells conduct a vital function in pathways leading to neurodegeneration [20]. In the normal condition, microglia block the nervous system by scavenging debris, regulating innate and adaptive immune responses, and killing pathogens. But, following brain injury or the beginning of a variety of neurodegenerative diseases such as, trauma, stroke, Alzheimer’s disease, Parkinson’s disease, and multiple sclerosis, microglial cells become stimulated and produce proinflammatory cytokines and neurotoxic materials, including tumor necrosis factor-α (TNF-α), interleukin-1β (IL-1β), IL-6, IL-12, nitric oxide (NO) [21]. Various proinflammatory mediators, including NO, PGE_2_, iNOS and COX-2 protein, and proinflammatory cytokines (IL-1β, IL-6, TNF-α, and IL-12), are major components of the neuroinflammatory condition in microglia [22]. The inhibition of these proinflammatory mediators and cytokines is a method of significant importance in the treatment of neuroinflammation. In this study, steppogenin suppressed the LPS-induced mRNA expression of proinflammatory cytokines, such as IL-1β, IL-6, TNF-α, and IL-12 (Figure 3), the production of NO and PGE_2_ (Figure 4A,B), and attenuated the protein expression of iNOS and COX-2, which induce NO and PGE_2_ respectively, in BV2 cells (Figure 4C). 

The activation of the NF-κB and MAPK pathways is reportedly associated with LPS-induced inflammation in BV2 microglial cells [23]. NF-κB is a transcriptional factor involved in the regulation of production or expression of proinflammatory mediators and cytokines [24]. MAPKs, including p38, ERK, and JNK, are also activated by various extracellular or intracellular stimuli, such as LPS; thus, these proteins are related to the inflammatory response [25]. Therefore, in this study, the effects of steppogenin on LPS-stimulated NF-κB and MAPKs activation were investigated. The results indicated that the pretreatment with steppogenin appeared to inactivate NF-κB by blocking the phosphorylation and degradation of IκB-α and the translocation of p65 and p50 dimer in BV2 cells (Figure 5A–C). Steppogenin also inhibited the DNA binding activity of p65 (Figure 5D), the localization of p50 into the nucleus in BV2 cells (Figure 5E), and the LPS-induced phosphorylation of JNK and p38 MAPK (Figure 6). These results showed that steppogenin appeared to exert antineuroinflammatory effects through the inactivation of the NF-κB, JNK, and p38 MAPK pathways. 

In this study, two types of microglial cells (BV2 and primary microglial cells) were used as in vitro models of neuroinflammation. BV2 cells are immortalized microglial cells from murine microglia, and have been used as an in vitro model of microglia in the field of neuroinflammation [26]. Primary microglial cells were isolated from the cerebral cortices of rats. Although these cells share similar properties in inflammatory conditions, BV2 cells do not have all the characteristics of microglial cells [11]. Therefore, the antineuroinflammatory effects of steppogenin in primary microglial cells were also investigated. The pretreatment of steppogenin attenuated the production of NO and the protein expression of iNOS and COX-2 in LPS-stimulated microglia (Figure 7). It was also shown that steppogenin reduced the LPS-induced mRNA expression of proinflammatory cytokines, such as IL-1β, IL-6, TNF-α, and IL-12, in primary microglial cells in a similar manner to that obsreved in BV2 cells (Figure 8). These observations in primary microglial cells provided evidence that steppogenin inactivated the NF-κB pathway (Figure 9). 

Previous studies have shown that various fractions or metabolites (e.g., cudratricusxanthone A) isolated from *C. tricuspidata* exert anti-inflammatory effects in RAW 264.7 macrophages [27,28,29]. The anti-inflammatory effect of a glycoprotein from *C. tricuspidata* in bisphenol A-treated HMC-1 cells has also been reported [30]. Steppogenin belongs to a class of flavanone that is widely distributed in *Moraceae* plants, such as *C. tricuspidata* and *Artocarpus heterophyllous*, and has been identified as a potent inhibitor of tyrosinase activity [31]. In addition, steppogenin-4′-*O*-β-d-glucoside exhibited hypoglycemic effects in a mouse model [32]. In this study, we demonstrated the antineuroinflammatory effect of steppogenin isolated from *C. tricuspidata* in LPS-stimulated microglia cells for the first time. Furthermore, we showed that steppogenin suppressed inducible NF-kB activation and the subsequent induction of proinflammatory mediators, such as NO, PGE2, IL-1β, IL-6, TNF-α, IL-12 and that it blocked the neuronal disorders including Alzheimer’s disease, Parkinson’s disease, and stroke [33,34,35].

## 4. Materials and Methods

### 4.1. Plant Materials and Isolation of steppogenin *(**1**)*

The root barks of *Cudrania tricuspidata* were purchased in May 2014 at Daerim Korean crude drug store, Kumsan, Chungnam Province, Korea, and identified by Dr. Kyu-Kwan Jang at the Botanical Garden, Wonkwang University. A voucher specimen (No. WP-2014-12) was deposited at the Herbarium of the College of Pharmacy, Wonkwang University (Iksan, Korea). Steppogenin (**1**) (Figure 1) was isolated from the methanol extract of *C. tricuspidata* (*Moraceae*) by various chromatographic methods and the structure was determined mainly through the analysis of MS and NMR data.

Steppogenin (**1**): ^1^H-NMR (DMSO-*d*_6_, 400 MHz) δ 5.61 (dd, *J* = 2.8, 13.2 Hz, H-2), 3.25 (dd, *J* = 13.2, 17.2 Hz, H-3a), 2.62 (dd, *J* = 2.8, 17.2 Hz, H-3b), 5.89 (br s, H-6, H-8), 6.37 (d, *J* = 1.6 Hz, H-3′), 6.29 (d, *J* = 1.6, 8.4 Hz, H-5′), 7.20 (d, *J* = 8.4 Hz, H-6′). ^13^C-NMR (DMSO-*d*_6_, 100 MHz) δ 74.0 (C-2), 41.3 (C-3), 197.0 (C-4), 163.7 (C-5), 95.9 (C-6), 166.7 (C-7), 95.1 (C-8), 163.6 (C-9), 101.9 (C-10), 115.6 (C-1′), 155.9 (C-2′), 102.6 (C-3′), 158.8 (C-4′), 106.7 (C-5′), 128.4 (C-6′) [7,36].

### 4.2. Chemicals and Reagents

Dulbecco’s modified Eagle’s medium (DMEM), fetal bovine serum (FBS), and other tissue culture reagents were purchased from Gibco BRL Co. (Grand Island, NY, USA). Lipopolysaccharides from *Escherichia coli* 055:B5 was purchased from Sigma-Aldrich (St. Louis, MO, USA). All other chemicals were obtained from Sigma Chemical Co. (St. Louis, MO, USA). Primary antibodies, including mouse/goat/rabbit anti-COX-2 (sc-1745), anti-iNOS (sc-650), anti-β-actin (sc-47778), anti-IкB-α (sc-371), anti-phospho-IкB-α (sc-8404), anti-p50 (sc-7178), anti-p65 (sc-8008), and anti-proliferating cell nuclear antigen (PCNA) (sc-7907), and secondary antibodies were purchased from Santa Cruz Biotechnology (Heidelberg, Germany). Anti-phospho-ERK (#9101), anti-ERK (#9102), anti-phospho-JNK (#9251), anti-JNK (#9252), anti-phospho-p38 (#9211), and anti-p38 (#9212) antibodies were obtained from Cell Signaling Technology (Danvers, MA, USA) [37].

### 4.3. Cell Culture and Viability Assay 

BV2 microglial cells were obtained from Prof. Hyun Park at Wonkwang University (Iksan, Korea). BV2 microglial cells were maintained at 5 × 10^6^ cells/dish (5 × 10^5^ cells/mL) in 100-mm diameter dishes in DMEM supplemented with 10% (*v/v*) heat-inactivated FBS, penicillin G (100 units/mL), streptomycin (100 μg/mL), and L-glutamine (2 mM), and incubated at 37 °C in a humidified atmosphere containing 5% CO_2_. To determine the cell viability, cells were plated in 96-well plates (2 × 10^4^ cells/well) and were incubated with 3-(4,5-dimethylthiazol-2-yl)-2,5-diphenyltetrazolium bromide (MTT) at a final concentration of 0.5 mg/mL for 4 h. The formed formazan salt was dissolved in acidified 2-propanol and the optical density of the solution was measured at 590 nm by using a microplate reader (Bio-Rad, Hercules, CA, USA). The optical density of the formazan formed in control (untreated) cells was considered to represent 100% viability. The assay was independently repeated three times [26,37,38,39]. 

### 4.4. Primary Microglial Culture

The cells dissociated from the cerebral hemispheres of the brains of one-day-old postnatal Sprague-Dawley rats were seeded in a T-75 flask (SPL Life Sciences, Pocheon, Korea) at a density of 1.2 × 10^6^ cells/mL in Dulbecco’s modified Eagle’s medium (DMEM; Gibco, Carlsbad, CA, USA) supplemented with 10% FBS and 1% penicillin-streptomycin. After 2 weeks, the microglia were detached by mild shaking and filtered through a cell strainer (BD Falcon, Bedford, MA, USA) to remove astrocytes. After centrifugation at 1000× *g* for 5 min, the cells were resuspended in fresh DMEM supplemented with 10% FBS and 1% penicillin-streptomycin and plated at a final density of 1.5 × 10^5^ cells/well on a 24-well culture plate. After 2 h, the medium was exchanged for DMEM supplemented with 5% FBS and 500 μM B27 (Gibco) [40].

### 4.5. Nitrite (NO Production) Determination

The nitrite concentration in the medium, an indicator of NO production, was measured by using the Griess reaction. Three independent assays were performed. An aliquot of the supernatant (100 μL) was mixed with an equal volume of Griess reagent (Solution A: 222488, Solution B: S438081; Sigma-Aldrich) and the absorbance of the mixture at 525 nm was determined by using an enzyme-linked immunosorbent assay (ELISA) plate reader (Bio-Rad model 680, Hercules, CA, USA) [37].

### 4.6. PGE_2_ Assay

The level of PGE_2_ present in each sample was determined by using a commercially available kit from R&D Systems (Minneapolis, MN, USA) in accordance with the manufacturer’s instructions. The assay was repeated independently three times. Briefly, BV2 microglial cells were cultured in 24-well plates, preincubated for 3 h with different concentrations of steppogenin, and then stimulated for 24 h with LPS. The cell culture supernatants were collected immediately after treatment and spun at 13,000× *g* for 2 min to remove particulate matter. The medium was added to a 96-well plate precoated with affinity-purified polyclonal antibodies specific for PGE_2_. An enzyme-linked polyclonal antibody specific for PGE_2_ was added to the wells, left to react for 24 h, and washed to remove any unbound antibody-enzyme reagent. After the addition of a substrate solution, the intensity of color produced was measured at 450 nm, which was proportional to the amount of PGE_2_ present [37].

### 4.7. Quantitative Real-Time Reverse Transcriptase PCR (qRT-PCR)

Total RNA was isolated from the cells by using Trizol (Invitrogen) in accordance with the manufacturer’s recommendations and quantified spectrophotometrically at 260 nm. Total RNA (1 μg) was reverse transcribed by using the High Capacity RNA-to-cDNA kit (Applied Biosystems, Carlsbad, CA, USA). The cDNA was then amplified by the SYBR Premix Ex Taq kit (TaKaRa Bio, Shiga, Japan) using a StepOnePlus Real-Time PCR system (Applied Biosystems). Briefly, each 20 μL reaction volume contained 10 μL SYBR Green PCR Master Mix, 0.8 μM of each primer, and diethyl pyrocarbonate (DEPC)-treated water. The primer sequences were designed by using PrimerQuest (Integrated DNA Technologies, Cambridge, MA, USA). The mouse primer sequences were 5′-CCA GAC CCT CAC ACT CAC AA-3′ (forward) and 5′-ACA AGG TAC AAC CCA TCG GC-3′ (reverse) for TNF-α; 5′-AAT TGG TCA TAG CCC GCA CT-3′ (forward) and 5′-AAG CAA TGT GCT GGT GCT TC-3′ (reverse) for IL-1β; 5′-ACT TCA CAA GTC GGA GGC TT-3′ (forward) and 5′-TGC AAG TGC ATC ATC GTT GT-3′ (reverse) for IL-6; 5′-AGT GAC ATG TGG AAT GGC GT-3′ (forward) and 5′-CAG TTC AAT GGG CAG GGT CT-3′ (reverse) for IL-12. The rat primer sequences were 5′-AGA GAC TTC CAG CCA GTT GC-3′ (forward) and 5′-AGT CTC CTC TCC GGA CTT GT-3′ (reverse) for IL-6; 5′-TGA CTT CAC CAT GGA ACC CG-3′ (forward) and 5′-GGA GAC TGC CCA TTC TCG AC-3′ (reverse) for IL-1β; 5′-CTC AGC GAG GAC ACC AAG GG-3′ (forward) and 5′-TGT ATG AGA GGG ACG GAA CCT-3 (reverse) primers for TNF-α, 5′-CCC CCA GAA TGT TTT GAC ACT-3′ (forward) and 5′-TGT GGG TGC TTC TGG AGT TT-3′ (reverse) for IL-12. The optimum conditions for PCR amplification of the cDNA were established by following the manufacturer’s instructions, and the data were analyzed by using StepOne software (Applied Biosystems, StepOne Software 2.0, Foster city, CA, USA) and the cycle number at the linear amplification threshold (Ct) values for the endogenous control gene (GAPDH) and the target gene were recorded. The relative gene expression (target gene expression normalized to the expression of the endogenous control gene) was calculated by using the comparative Ct method (2^−ΔΔ*C*t^). The analysis was repeated independently three times [37].

### 4.8. Preparation of Cytosolic and Nuclear Fractions

BV2 and primary rat microglial cells were homogenized in M-PER™ Mammalian Protein Extraction Buffer (1:20, *w/v*) (Pierce Biotechnology, Rockford, IL, USA) containing freshly added protease inhibitor cocktail I (EMD Biosciences, San Diego, CA, USA) and 1 mM phenylmethylsulfonylfluoride (PMSF). The cytosolic fraction of the cells was prepared by centrifugation at 16,000× *g* for 5 min at 4 °C. The nuclear and cytoplasmic cell extracts were prepared with NE-PER^®^ nuclear and cytoplasmic extraction reagents (Pierce Biotechnology, Rockford, IL, USA), respectively [37].

### 4.9. DNA Binding Activity of NF-κB

BV2 microglial cells were pretreated for 3 h with the indicated concentrations of steppogenin and then stimulated for 1 h with LPS (1 μg/mL). The DNA-binding activity of NF-κB in the nuclear extracts was measured by using the TransAM^®^ kit (Active Motif, Carlsbad, CA, USA) in accordance with the manufacturer’s instructions. The assay was repeated independently three times [37].

### 4.10. Western Blot Analysis

BV2 and primary rat microglial cells were harvested and pelleted by centrifugation at 16,000 rpm for 15 min. The pelleted cells were washed with phosphate-buffered saline (PBS) and lysed with 20 mM Tris-HCl buffer (pH 7.4) containing a protease inhibitor mixture (0.1 mM PMSF, 5 mg/mL aprotinin, 5 mg/mL pepstatin A, and 1 mg/mL chymostatin). The protein concentration was determined by using a Lowry protein assay kit (P5626; Sigma-Aldrich), and an equal amount of protein (30 µg) from each sample was resolved by using 7.5% or 12% sodium dodecyl sulfate-polyacrylamide gel electrophoresis (SDS-PAGE) and then electrophoretically transferred onto a Hybond™ enhanced chemiluminescence nitrocellulose membrane (Bio-Rad). The membrane was blocked with 5% (*w/v*) skim milk and sequentially incubated with the primary antibody (Santa Cruz Biotechnology, Santa Cruz, CA, USA) and the horseradish peroxidase-conjugated secondary antibody followed by ECL detection (Amersham Pharmacia Biotech, Piscataway, NJ, USA). The intensities of band signals were quantified densitometrically by using ImageJ software (National Institutes of Health, Bethesda, MD, USA). Molecular weight markers and the internal standards, β-actin and PCNA, were also run on the gel. The analysis was repeated independently three times [38].

### 4.11. NF-κB Localization and Immunofluorescence

BV2 and primary rat microglial cells were cultured on Lab-Tek II chamber slides and treated as described in the figure legends. The cells were treated with 80.0 μM steppogenin for 1 h, fixed in formalin, permeabilized with cold acetone, and then probed with a primary antibody against NF-κB and a fluorescein Isothiocyanate (FITC)-labeled secondary antibody (Alexa Fluor 488; Invitrogen, Carlsbad, CA, USA). To visualize the nuclei, the cells were treated with DAPI (1 μg/mL) for 30 min, washed with PBS for 5 min, and treated with 50 μL VectaShield (Vector Laboratories, Burlingame, CA, USA). The stained cells were visualized and photographed by using a Zeiss fluorescence microscope (Provis AX70; Olympus Optical Co., Tokyo, Japan) [38].

### 4.12. Statistical Analysis

The data are expressed as the mean ± SD of at least three independent experiments. To compare three or more groups, one-way analysis of variance (ANOVA) followed by Tukey’s multiple comparison tests was carried out. Data were analyzed by using GraphPad Prism software, version 3.03 (GraphPad Software Inc., San Diego, CA, USA) [40].

## 5. Conclusions

**Figure 10:**
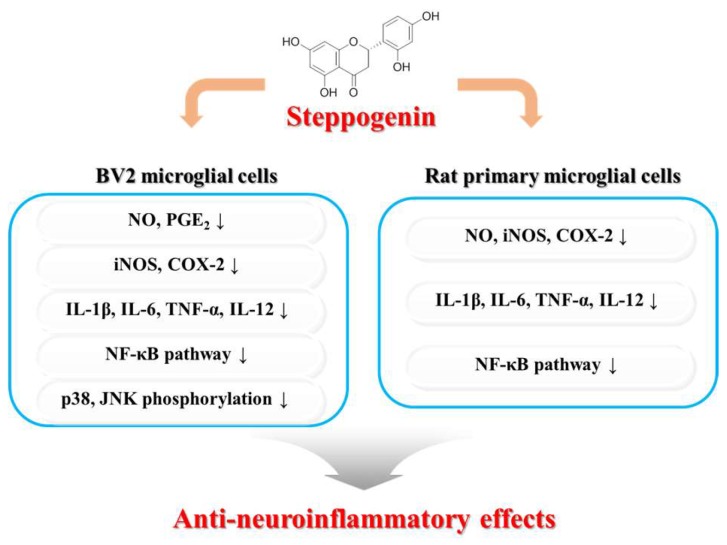
Schematic diagram showing the relationship of steppogenin (**1**) and NF-κB/inflammation/MAPK damage after LPS in BV2 microglial and rat primary microglial cells.

In conclusion, this study revealed that steppogenin isolated from *Cudrania tricuspidata* showed potent antineuroinflammatory effects in BV2 and primary microglial cells. These effects were due to the ability of steppogenin to inactivate the NF-κB, JNK, and p38 pathways, to inhibit the protein expression of iNOS, COX-2, and proinflammatory cytokines, and to suppress the production of NO and PGE_2_. Thus, steppogenin could be a potent drug candidate for the treatment of various neurodegenerative diseases that arise from neuroinflammation (Figure 10).
