# Correction: Kim, D.-C.; et al. Steppogenin Isolated from *Cudrania tricuspidata* Shows Antineuroinflammatory Effects via NF-κB and MAPK Pathways in LPS-Stimulated BV2 and Primary Rat Microglial Cells. *Molecules* 2017, *22*, 2130

**DOI:** 10.3390/molecules23061244

**Published:** 2018-05-23

**Authors:** Dong-Cheol Kim, Tran Hong Quang, Hyuncheol Oh, Youn-Chul Kim

**Affiliations:** 1 Institute of Pharmaceutical Research and Development, College of Pharmacy, Wonkwang University, Iksan 54538, Korea; kimman07@hanmail.net (D.-C.K.); hoh@wku.ac.kr (H.O.); 2 Institute of Marine Biochemistry, Vietnam Academy of Science and Technology (VAST), 18 Hoang Quoc Viet, Caugiay, Hanoi 122102, Vietnam; quangth2004@yahoo.com; 3 Hanbang Cardio-Renal Syndrome Research Center, Wonkwang University, Iksan 54538, Korea

The author wishes to make the following correction to this paper [1]. After comparing the published figures with their raw data, we found that the second band (Nuclear-p50) in Figure 5C had been attached incorrectly. Therefore, we would like to replace Figure 5C:
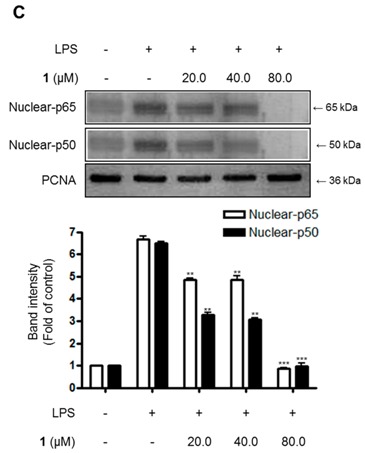
 with 
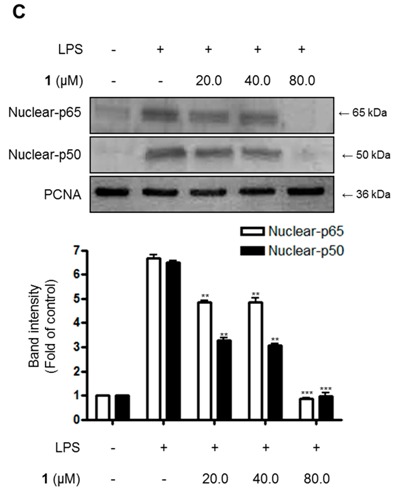


The authors would like to apologize for any inconvenience caused to the readers by these changes.
